# The launch of the Pacific vector network: connecting Pacific Island Countries and areas to prevent and control vector-borne diseases

**DOI:** 10.1186/s13071-025-06760-9

**Published:** 2025-03-22

**Authors:** Limb K. Hapairai, Salanieta T. Saketa, Amandeep Singh, Rosanna Y. Rabago, Amanda K. Murphy, Tessa B. Knox, Nuha Mahmoud, Emi Chutaro, Anna Drexler, Pacific Vector Network contributors

**Affiliations:** 1 Pacific Island Health Officers’ Association (PIHOA), Honolulu, HI USA; 2 The Pacific Community (SPC), Suva, Fiji; 3 Division of Pacific Technical Support, World Health Organization (WHO), Suva, Fiji; 4 James Cook University (JCU), Cairns, Australia; 5 U.S. Centers for Disease Control and Prevention (CDC), Ft. Collins, CO USA

**Keywords:** Pacific, Vector, Network, Vector-borne disease

## Abstract

The Pacific Island Health Officers’ Association, the World Health Organization, and the Pacific Community co-organized the launch of the Pacific Vector Network (PVN) to address challenges posed by mosquito-borne diseases, including dengue fever, Zika virus disease, chikungunya, malaria, and lymphatic filariasis. The PVN was created as a new initiative under the Pacific Public Health Surveillance Network (PPHSN). This launch was a critical step in the build-up to PVN as a full-service network of PPHSN in the coming years. The Pacific Island Countries and areas (PIC)-led network comprises vector management leadership, officers, and technical partners dedicated to supporting information-sharing to promote evidence-based collective action and innovation. The setup of a Technical Working Body to ensure governance and to steer forward the work of the network was a key deliverable. This manuscript describes the proceedings and discussions of PIC representatives and several regional partners at the inaugural PVN meeting held 5–7 June 2023 in Hawai’i, USA.

**Graphical Abstract:**

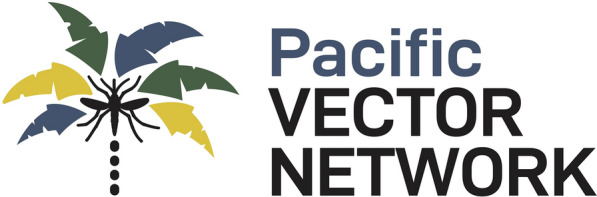

## Background

The Pacific region is home to 9 million people who continue to be threatened or afflicted by multiple vector-borne diseases (VBDs) [1]. The region harbors 423 mosquito species, some transmitting diseases such as dengue fever, Zika virus fever, chikungunya, and Japanese encephalitis, or parasitic diseases such as malaria and lymphatic filariasis [2]. In recent years, VBD outbreaks have increased in intensity and frequency throughout the Pacific region and globally, with 19 of 22 Pacific Island Countries and areas (PICs) declaring at least one arboviral disease outbreak between 2018 and 2020, compared with 14 of 22 PICs between 2012 and 2014 [3]. These strain fragile health systems and often pose a risk to human health throughout the region, causing a surge in cases [3]. However, PICs lack capacities and systems to routinely monitor and respond effectively to VBD outbreaks, each time overwhelming the PICs’ available public health resources. Consequently, strengthening vector control programs to reduce the overall burden of VBDs has become a top priority across the Pacific [4], aligning with the World Health Organization (WHO) Global Vector Control Response (GVCR) 2017–2030 [5].

Although epidemiological surveillance of VBDs has improved in the Pacific region over the last decade [6], many PICs struggle to maintain an effective VBD preparedness and response program, which requires a multidisciplinary and multisectoral approach [7]. In 2019, the Pacific Community (SPC) and WHO convened a consultation with PICs and partners, during which the countries recommended the creation of a network for the Pacific to support capacity strengthening in line with the GVCR 2017–2030 [8]. The Pacific Public Health Surveillance Network (PPHSN) is a voluntary network of countries and organizations dedicated to the promotion of public health surveillance and appropriate response to the health challenges of American Samoa, Cook Islands, Federated States of Micronesia (FSM), Fiji, French Polynesia, Guam, Kiribati, Marshall Islands, Nauru, New Caledonia, Niue, Northern Mariana Islands, Palau, Papua New Guinea (PNG), Pitcairn Islands, Samoa, Solomon Islands, Tokelau, Tonga, Tuvalu, Vanuatu, and Wallis and Futuna. In October 2022, the 22 PICs that comprise PPHSN approved a new country-led initiative for vector management under the PPHSN. This decision mandated that SPC, WHO, and the Pacific Island Health Officers’ Association (PIHOA) jointly provide secretariat support to coordinate the new network. The network was named the Pacific Vector Network (PVN) to emphasize preparedness and response to VBD efforts.

## Meeting report

**Fig. 1:**
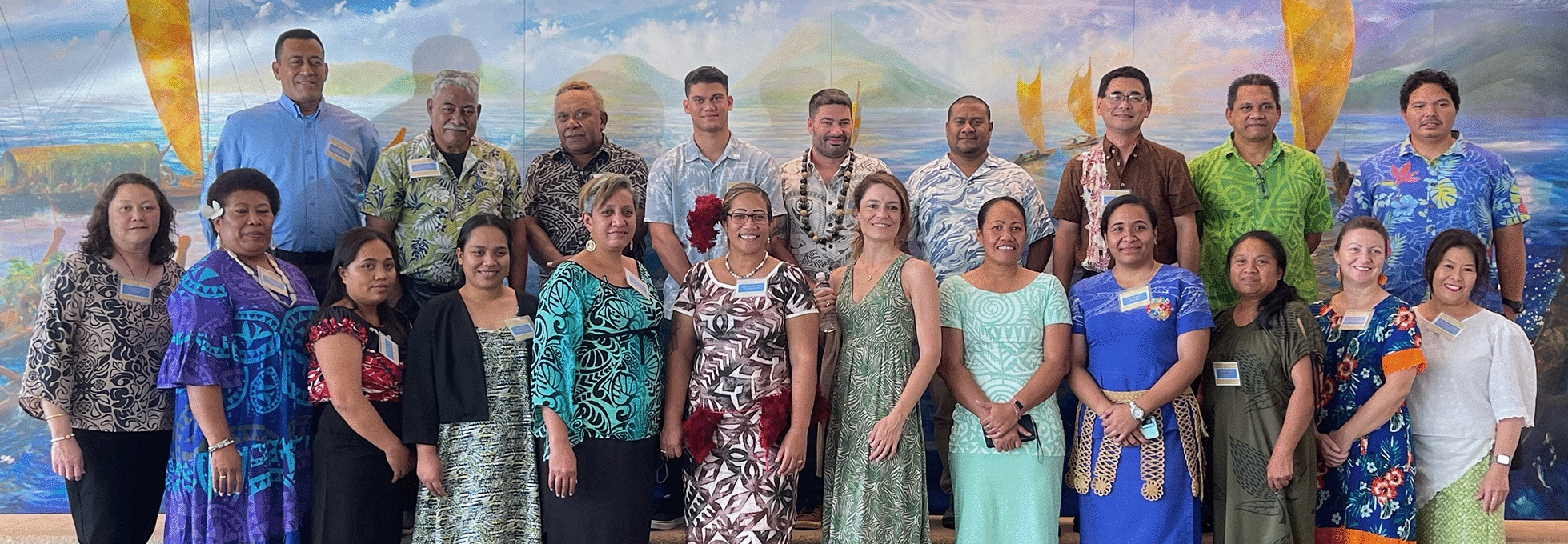
Participants at the inaugural Pacific Vector Network meeting in Honolulu, Hawai’i, USA, 5–7 June 2023

**Fig. 2:**
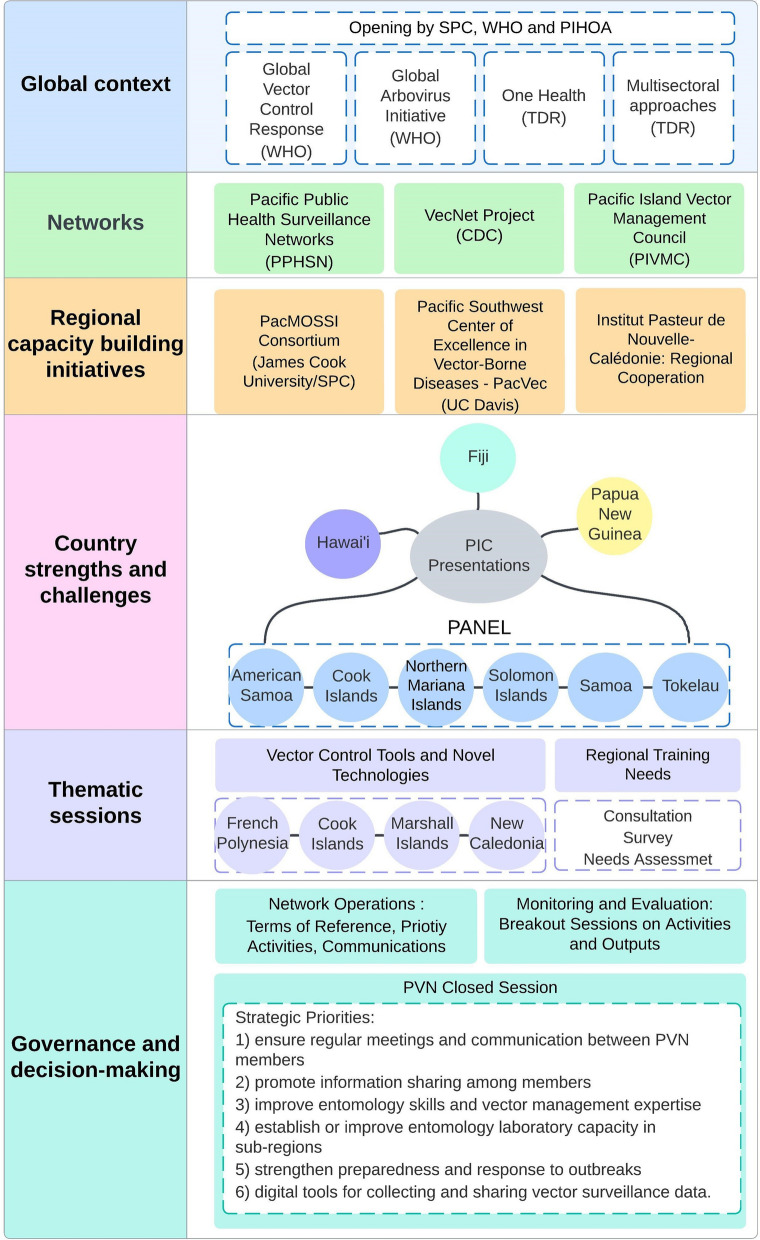
Meeting framework for the inaugural Pacific Vector Network meeting

Herein, we describe the proceeding of the inaugural meeting held on 5–7 June 2023 in Hawai’i, USA. We demonstrate how the PVN connects VBD control practitioners, including PICs’ environmental health officers, entomologists, epidemiologists, researchers, scientists, and academics, to consult, enhance, and promote an effective locally adapted sustainable vector management. The meeting was attended in person by representatives of 16 PICs and 10 regional partner institutions (Fig. 1). The meeting was held in a hybrid format to maximize the participation of member countries and partner institutions in support of the goal of the PVN. The meeting was organized into sections: global context, networks, regional capacity-building initiatives, country strengths and challenges, thematic sessions, and governance and decision-making (Fig. 2).

The meeting was opened with welcoming remarks from Minister Gaafar Uherbelau (PIHOA Board President and Palau Minister of Health and Human Services), Dr. Salanieta Saketa (SPC), and Dr. Nuha Mahmoud (WHO). Plenary presentations were given by Dr. Raman Velayudhan (WHO) on the status of WHO’s GVCR and Global Arbovirus Initiative and Dr. Florence Fouque (Tropical Disease Research (TDR)/WHO) on TDR research priorities and ongoing vector control and multisectorial activities. Several PIC representatives raised questions on novel vector control intervention acceptability, community participation, training for implementation, and mechanisms for PICs to access new technologies, such as the sterile insect technique (SIT) and *Wolbachia*-based strategies.

To highlight other regional networks, Dr. Saketa provided an overview of PPHSN and its six service networks, which support the surveillance and control of infectious diseases across PICs. Dr. Amanda Murphy (WHO) outlined the background and rationale for establishing the PVN indicated through PIC consultations [4], Dr. Anna Drexler (US Centers for Disease Control and Prevention, CDC) outlined global networks supported by the CDC through their VecNet project [9], and Mr. Thomas Nadeau (Guam Department of Public Health and Social Services, Division of Environmental Health) described the functions and experience of the Pacific Island Vector Management Council (PIVMC) [10] serving the US-affiliated Pacific Islands of American Samoa, Guam, FSM, Northern Mariana Islands, Marshall Islands, and Palau. General discussion points raised by PIC representatives included: the need to recognize EH as a discipline in the region and emphasize the need for strengthening communication within PIC EH teams and other departments (i.e., epidemiology and lab teams); building in-country entomology capacity; strengthening technical partner support in-country, rather than remotely; supporting vector management programs; establishing and implementing outbreak response plans; and prioritizing the importance for routine vector surveillance across PICs.

To feature regional capacity-building projects, Dr. Tanya Russell (James Cook University) provided an overview of support provided to PICs by the Pacific Mosquito Surveillance Strengthening for Impact (PacMOSSI) consortium to complete vector control needs assessments, training programs, and capacity-building activities, emphasizing the importance of collaboration and regional partnerships established across PICs and partners [11]. Dr. Christopher Barker (University of California-Davis) presented the Pacific Southwest Center of Excellence in Vector-Borne Diseases’ (PacVec) training activities and the VDB Surveillance Gateway (PacSurv) operating in the USAPIs [12]. Dr. Nicholas Pouquet (Institut Pasteur de Nouvelle-Calédonie) outlined the mission and work of Institut Pasteur, emphasizing its contributions in research, education, development of new facilities, public health initiatives, and regional cooperation in Fiji, Vanuatu, and Wallis and Futuna. Discussion points raised by PIC representatives focused on the need to coordinate capacity-building activities, leverage opportunities, standardize strategies, design a Pacific-wide monitoring framework, and promote practical training to improve the skills and retention of vector management staff.

Country reports from vector managers from Hawai’i, PNG, and Fiji were discussed. Mr. Matthew Kurano (Hawai’i Department of Health) outlined the historical events that shaped Hawaii’s vector management program operations, challenges, and needs. Mr. Elias Omera (PNG Institute of Medical Research) presented on malaria incidence in PNG and the NatNat project collaboration aiming to build local entomology infrastructure, provide training and testing, and evaluate vector control tools. Mr. Vimal Deo (Fiji Ministry of Health and Medical Services) presented an overview of vector management activities, surveillance data management, insecticide use, and post-intervention monitoring of *Aedes aegypti* for *Wolbachia* in Fiji. Presentations were then followed by convening a panel of representatives from American Samoa, Cook Islands, Northern Mariana Islands, Samoa, Tokelau, and Solomon Islands, who were asked to share the strengths and challenges of implementing vector management in their countries. Strengths identified included several PICs conducting routine surveillance and reporting, committed teams despite being stretched, developing national strategies and standard operating procedures (SOPs) for VBDs, and building strong structures to facilitate community participation. Challenges included inconsistent funding availability; competing priorities within and among government departments and stakeholders; a lack of trained staff, equipment, and supplies; and limited access to technical expertise in-country.

Two thematic sessions were held, with the first focused on vector control tools and novel technologies. Dr. Anna Drexler provided an overview of different types of novel vector control tools under development globally, including replacement and suppression strategies for *Aedes* and *Culex* species. Mr. Hitinui Teinaore (Directorate of Health, French Polynesia) and Dr. Hervé Bossin (Institut Louis Malardé, French Polynesia) presented on the implementation in French Polynesia of a SIT and *Wolbachia* approach to control of *Ae. aegypti* and *Aedes polynesiensis*. Ms. Earlynta Chutaro (Republic of Marshall Islands Ministry of Health and Human Services) described the Ma Nam Ne project to suppress *Ae. aegypti* using genetic manipulation technology in the Marshall Islands. Ms. Florie Cheilan (New Caledonia Direction of Health and Social Affairs) and Dr. Nicholas Pouquet presented the findings from implementing the *Wolbachia* replacement strategy against *Ae. aegpyti* in New Caledonia. The PIC representatives raised questions on novel approach risks, safety, criteria, cost, and the possibility of establishing a regional entomology laboratory to support regional initiatives, including novel technologies.

Dr. Amanda Murphy moderated the second thematic session on regional training needs, presenting the outcomes of consultations in 2022 and a survey of 12 PICs on enhancing coordination and information sharing for vector management. She highlighted key types of information to be shared, activities to be undertaken, and communication methods. Country priorities included improving skills and capacity, strengthening preparedness and response to outbreaks, and using digital tools. Following this, breakout groups with PICs and partners discussed priority training needs, the challenges of maintaining a trained workforce, and opportunities that can be leveraged in the Pacific regions. The groups emphasized the need for multisectoral synergies [e.g., points of entry management, Environmental Health (EH), invasive species, and biosecurity]; disaster preparedness; entomology training with a preference for on-island instruction; and sharing among PICs vector management information, SOPs, and lessons learned from routine and enhanced surveillance and outbreak response activities.

Dr. Murphy’s presentation on network operations underscored the priority activities, communications, and key aspects of the draft PVN Terms of Reference (ToR), scheduled for deliberation by members on day 3 of the meeting. The group identified the potential for the PVN to facilitate information sharing among PICs and partners, emphasizing the need for trust-building to enable data sharing and the importance of starting small by evaluating existing data. The group recognized that limitations in testing capacity and reliance on out-of-country testing remained a significant challenge.

Mr. Gerald Jacobson (Universidad del Valle in Guatemala) delivered a comprehensive presentation on monitoring and evaluation (M&E) during the session, followed by breakout activities and group discussions. The presentation highlighted the purpose, activities, and application of M&E in various networks. Mr. Jacobson also provided an overview of the essential components that should be included in an M&E plan. To further enhance understanding, a breakout activity was conducted to define activities, identify potential milestones, and brainstorm evaluation methods. The participants provided feedback on M&E planning indicators for strengthening vector management, formalizing the exchange of knowledge and resources between the PICs and ensuring the sustainability of the PVN. Additionally, they explored the relationship between M&E and early warning systems (EWS).

Additionally, PIC representatives met in a closed session without partners present to discuss the ToRs, strategic plan, priority activities, and next steps. Later, in the open session, the PICs shared their conclusions. They identified six priorities for the PVN as follows: (1) ensure regular meetings and communication between PVN members, (2) promote information sharing among members, (3) improve entomology skills and vector management expertise, (4) establish or improve entomology laboratory capacity in each subregion, (5) strengthen preparedness and response to outbreaks of vector-borne diseases, and (6) support the use of digital tools for collecting and sharing vector surveillance data.

**Fig. 3:**
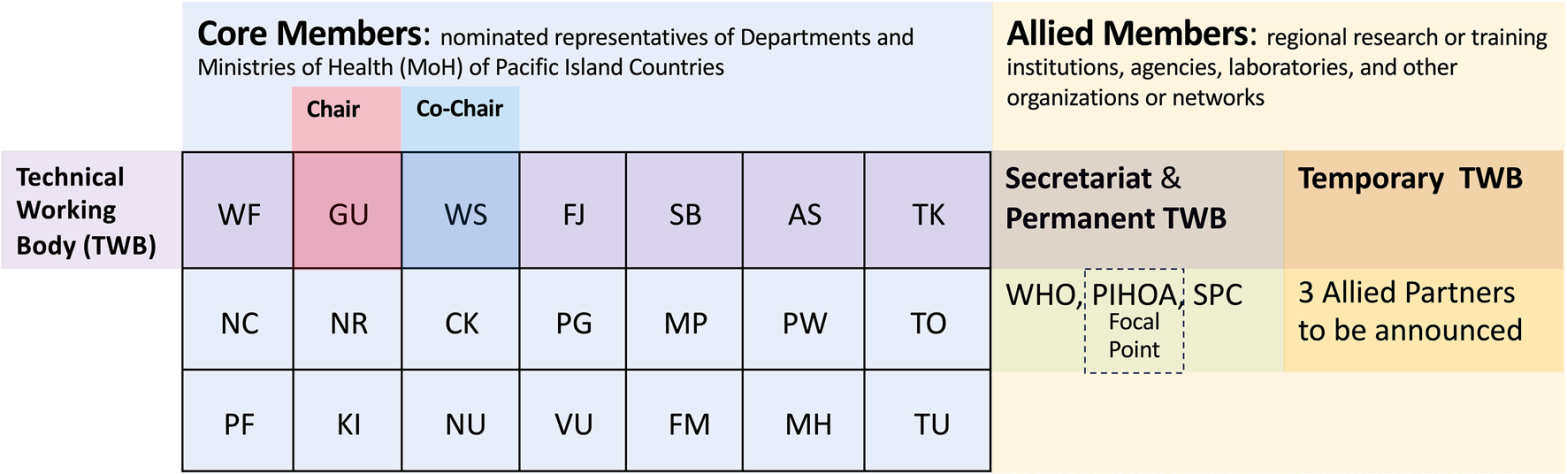
Governance of the Pacific Vector Network. Core members include representatives of American Samoa (AS), Cook Islands (CK), Federated States of Micronesia (FM), Fiji (FJ), French Polynesia (FP), Guam (GU), Kiribati (KI), Marshall Islands (MH), Nauru (NR), New Caledonia (NC), Niue (NU), Northern Mariana Islands (MP), Palau, Papua New Guinea (PG), Samoa (WS), Solomon Islands (SB), Tokelau (TK), Tonga (TO), Tuvalu (TU), Vanuatu (VU), and Wallis and Futuna (WF). Pitcairn Islands was added recently. The Secretariat consists of the World Health Organization (WHO), the Pacific Island Health Officers Association (PIHOA), and the Pacific Community (SPC)

Representatives from the 16 PICs in attendance engaged in a thorough review and refinement of draft PVN ToRs for endorsement [13]. The network consists of 22 PICs, of which 7 were nominated as the PVN Technical Working Body (TWB), which serves as the governing and decision-making entity for network activities. These representatives include Guam (Chair), Samoa (Co-chair), American Samoa, Fiji, Solomon Islands, Tokelau, and Wallis and Futuna (Fig. 3). The remaining PICs will participate in the TWB during their designated rotation period. The PVN TWB also includes three permanent allied partners: the Secretariat of PIHOA, SPC, and WHO, and up to three temporary allied partners. Candidate allied partners must apply to the Secretariat with applications reviewed by the TWB for selection.

Lastly, it was decided that the second annual PVN meeting would be held in Guam in July 2024.

## Conclusions

PICs face significant challenges in addressing VBDs, including increasing outbreak intensity and frequency, strained health systems, and implementation and resource gaps in vector management programs. The PVN was established to build regional capacity through collaboration, knowledge-sharing, and technical exchange. The inaugural meeting emphasized the need for regular communication, information sharing, and working together to strengthen vector management. Key priorities for the PVN, as identified by PIC representatives, were improving entomology skills, enhancing entomological laboratory capacity, and strengthening preparedness and response mechanisms. Recommendations included developing a long-term strategic plan including an M&E framework, a communications strategy, implementing training programs in coordination with other partners, and upgrading regional laboratories. By facilitating information sharing, standardizing strategies, and enabling more effective prevention and response to VBD outbreaks, this collaborative effort aims to reduce the burden of VBDs and improve overall public health resilience in the Pacific region.

## Data Availability

No datasets were generated or analyzed during the current study.
